# Acute and chronic fluid misdirection syndrome: pathophysiology and treatment

**DOI:** 10.1007/s00417-017-3837-0

**Published:** 2017-11-06

**Authors:** Andrzej Grzybowski, Piotr Kanclerz

**Affiliations:** 1 Department of Ophthalmology, University of Warmia and Mazury, Olsztyn, Poland; 2 Institute for Research in Ophthalmology, Foundation for Ophthalmology Development, 60-554 Gorczyczewskiego 2/3, Poznan, Poland; 3 Department of Ophthalmology, Medical University of Gdańsk, Gdańsk, Poland

**Keywords:** Cataract, Glaucoma, angle-closure, Trabeculectomy, Vitrectomy, Vitreous body

## Abstract

**Purpose:**

To summarize our current understanding of the specific pathogenic mechanisms of the *fluid misdirection syndrome* and possible treatment methods.

**Methods:**

We used the PubMed web platform to find relevant studies using the following keywords: *infusion misdirection syndrome, aqueous misdirection syndrome, ciliary block, ciliovitreal block, capsular block, intraoperative fluid misdirection, subcapsular fluid entrapment, acute intraoperative rock-hard eye syndrome, positive vitreous pressure glaucoma*, and *malignant glaucoma*. Other publications were also considered as a potential source of information when referenced in relevant articles.

**Results:**

We collected and analyzed 55 articles dated from 1951 to 2016. *Acute intraoperative rock-hard eye syndrome* is characterized by a very shallow anterior chamber with the absence of suprachoroidal effusion or hemorrhage and no noticeable pathology of the iris–lens diaphragm. It usually occurs during uneventful phacoemulsification, particularly in hyperopic eyes. The pathophysiology of *acute fluid misdirection syndrome* is based on inappropriate movement of balanced salt solution via the zonular fibers. This syndrome has also been described as occurring from hours to months, or years, after the initial surgery. The pathophysiology of *malignant glaucoma* is based on similar mechanisms of cilio-lenticular block of aqueous flow leading to the misdirection of aqueous posteriorly into or besides the vitreous gel. Faced with these situations, vitreous decompression is required, preferably with hyaloido-capsulo-iridectomy. In phakic eyes, concomitant cataract extraction would be desirable.

**Conclusions:**

We believe both of these clinical conditions should be considered as one syndrome. We suggest the term *acute fluid misdirection syndrome* for the cascade of events during phacoemulsification surgery. *Chronic fluid misdirection syndrome* better describes the nature of *malignant glaucoma*.

## Introduction

The *fluid misdirection syndrome* is a serious threat in anterior segment surgery. It is notoriously difficult to treat, and carries a generally uncertain prognosis for long-term control of intraocular pressure. Our understanding of pathophysiology, risk factors, accurate diagnosis, and treatment is hampered by insufficient uniform clinical case clarification. Previously proposed definitions for *fluid misdirection syndrome* were nonspecific and lacking a comprehensive view. It has been also reported under different names, including *infusion misdirection syndrome, aqueous misdirection syndrome, capsular block, ciliovitreal block, ciliary block, intraoperative fluid misdirection, subcapsular fluid entrapment, acute intraoperative rock-hard eye syndrome, positive vitreous pressure glaucoma*, and *malignant glaucoma*. Currently, years after the initial description by von Graefe in 1869 [1], we now know much more about this uncommon syndrome, but its characterization still remains incomplete.

This review summarizes our current understanding of the specific pathogenic mechanisms of this rare syndrome and possible treatment methods. Certainly it will assist in identification of risk factors and make it possible to establish an effective treatment of this dangerous syndrome.

## Methodology

We used the PubMed web platform to find prospective or retrospective studies and case reports.

Our keywords have been strictly defined: *infusion misdirection syndrome, aqueous misdirection syndrome, ciliary block, ciliovitreal block, capsular block, intraoperative fluid misdirection, subcapsular fluid entrapment, acute intraoperative rock-hard eye syndrome, positive vitreous pressure glaucoma*. Other publications were also considered as a potential source of information when referenced in relevant articles.

We selected English language articles and divided our reports into two groups: intraoperative complications (Table 1) and postoperative complications (Table 2). Common risk factors for *fluid misdirection syndrome* are presented in Table 3.

**Table 1:** Clinical manifestations of *acute fluid misdirection syndrome*

Article	Suggested name of syndrome	No. of eyes (incidence)	Performed procedure	Risk factors	Time of occurrence during surgery	Presumed pathomechanism	Suggested treatment	Outcome (follow-up period)
Kam et al. 2016 [2]	Acute intraoperative rock-hard eye syndrome	Two eyes (2/736 phaco procedures)	Phaco + IOL	N/A	During surgery (not described)	Freeing tenacious cortex, direct irrigation with a straight or a curved cannula of the inferior or superior capsular bag fornix, may contribute to finding materials in the vitreous. When repeated with a single, potentially powerful jet of balanced salt irrigated through a syringe and a cannula, may lead to transgression of the zonular apparatus by the balanced salt solution and small pieces of lens material.	Emergent pars plana needle aspiration of retrolenticular fluid with a 23-gauge needle	Good — AC deepening
Lau et al. 2014 [3]	Acute intraoperative rock-hard eye syndrome	Six eyes (6/413 phaco procedures)	Phaco + IOL	- Higher levels of AC irrigation - Hyperopia	Not only from the phacoemulsification but also from hydrodissection, hydrodelineation, and irrigation of the posterior capsule and equatorial cortex with a hydrodissection cannula.	Residual cortical fiber irrigation maneuver (when residual cortical fibers are being removed, the narrow stream of balanced salt solution generated by the narrow hydrodissection cannula tip may distort the posterior capsule locally) resulting in localized anterior displacement of the contiguous posterior capsule, creating a subcapsular or vitreal space through which the balanced salt solution may move via the zonular fibers.	Emergent pars plana (3 mm from limbus) needle aspiration of retrolenticular fluid with a 23-gauge needle	Good post-op visual acuity and normal IOP. Complications: - mild vitreous hemorrhage (one eye) - temporal arteritis (one eye 1 month post-op)
Mackool et al. 1993 [4]	Infusion misdirection syndrome	N/A	Phaco + IOL	- Exfoliation - Dense/brunescent cataract	During surgery, especially at the time of removal of the last nuclear pieces	Aqueous can be directed posteriorly behind an intact lens and cause chamber shallowing: - when fluid passes through an opening in the posterior capsule or the equatorial region (especially likely after can-opener capsulotomy or radial extension of capsulorhexis), permitting direct access to the retrocapsular space - in the presence of an intact capsule (intact rim by capsulorhexis) fluid passes through the region of the zonular fibers. More commonly in association with lax zonular fibers	- Viscoadaptive agent to the AC - Pars plana decompression	N/A
Olson et al. 1994 [5]	Subcapsular fluid entrapment	Five eyes	Penetrating keratoplasty + ECCE + IOL (two eyes) phaco + IOL (three eyes)	- Open sky I/A - Zonular dialysis	During surgery	- Irrigation anterior to the AC flap - Zonular dehiscence	- Dry aspiration (three eyes) - Pars plana paracentesis (one eye) - Spontaneous central capsule rupture (one eye)	Good — AC deepening Paracentesis unsuccessful in one eye — IOL implanted 2 months post-op
Dewey 2011 [6]	Intraoperative fluid misdirection	Three eyes	Phaco + IOL	- Coughing - Short axial length - Significantly intumescent lens	During surgery	N/A	- Viscoadaptive agents to the AC and dry aspiration (mild cases) - Sharp needle decompression, or automated vitrectomy handpiece to avoid potential vitreous traction (severe cases) - Benzonatate capsules (Tessalon Perles) for patients with a history of coughing, “sinus drainage,” chronic obstructive pulmonary disease, bronchitis, asthma, sleep apnea, snoring, use of supplemental oxygen, difficulty breathing in the supine position for any reason, and at the preoperative nurse’s suspicion.	Good — AC deepening
Grzybowski et al. 2014 [7]	Acute / chronic aqueous misdirection syndrome	N/A	Phaco + IOL	N/A	During surgery - toward the end of irrigation/aspiration	Inappropriate “movement of the balanced salt solution via the zonular fibers” triggered by the unconventional use of the residual cortical fiber irrigation maneuver	Pars plana decompression with vitreous cutter	Good — AC deepening
Wu et al. 2016 [8]	Malignant glaucoma	Thirty-eight eyes (of 1432 eyes undergoing anti-glaucoma surgical treatment for primary angle closure glaucoma)	- After trabeculectomy (32 eyes — 84.2%) - during trabeculectomy (four eyes — 10.5%) - after combined glaucoma/cataract surgery (two eyes — 5.3%)		During or after surgery	1) Ciliolenticular block or anterior hyaloid obstruction 2) Slackness of lens zonules 3) Severe postoperative inflammation 4) Non ciliary block. It was observed that those patients who developed malignant glaucoma during surgery all showed a high stress level and poor operative cooperation. We speculated that such status caused constriction of extraocular muscles pressing forward, bringing about the increase of ocular venous pressure.		

Abbreviations used: AC, anterior chamber; ECCE + IOL, extracapsular cataract extraction with intraocular lens implantation; I/A, irrigation/aspiration; IOL, intraocular lens; IOP, intraocular pressure; phaco + IOL, phacoemulsification with intraocular lens implantation

**Table 2:** Clinical manifestations of *chronic fluid misdirection syndrome*

Article	Suggested name of syndrome	No. of eyes (incidence)	Performed procedure	Risk factors	Time of occurrence related to time of surgery	Presumed pathomechanism	Suggested treatment	Outcome (follow-up period)
Little et al. 1993 [9]	Malignant glaucoma, ciliolenticular block, ciliovitreal block, iridovitreal block, aqueous misdirection or aqueous diversion syndrome	Seven eyes (7/ 12,000 surgeries)	- ECCE + IOL (four eyes) - ECCE + IOL + trabeculectomy (two eyes) - trabeculectomy (one pseudophakic eye)	N/A	1 week–7 months	Cilio-lenticular block of aqueous flow leading to the misdirection of aqueous posteriorly into or in front of the vitreous gel leading to the characteristic diffuse shallowing of the AC accompanied by a precipitous rise in IOP.	Nd:YAG laser capsulotomy and/or vitreolysis	IOP stabilized in five of seven eyes
Sharma et al. 2006 [10]	Aqueous misdirection syndrome	Five eyes	- Trabeculectomy (one eye) - Redo-trabeculectomy (one eye) - YAG LPI (one eye) - spontaneous, following branch retinal vein occlusion, NdYAG LPI, trabeculectomy, cyclodiode (one eye) - N/A (though phaco-emulsification of the cataract in the fellow eye had resulted in a severe postoperative aqueous misdirection syndrome)	Hyperopia	1 day–3 months	Blockage of anterior aqueous flow at the level of the ciliary body combined with an inherent impermeability defect in the anterior hyaloid. Abnormal anatomic relationship between the ciliary body, anterior hyaloid, and lens in hyperopic eyes. A forward displacement of the anterior vitreous with apposition of the anterior hyaloid face against the lens and ciliary body prevents the normal anterior flow of aqueous humor. Subsequent misdirection of aqueous flow posteriorly and its accumulation in the posterior segment, with increasing posterior pressure, results in anterior displacement of the iris–lens diaphragm, axial and peripheral AC flattening, and secondary angle closure	Core vitrectomy - phacoemulsification - vitrectomy with iridozonulohyaloidectomy	IOP stabilization with topical hypotensive agents Complications: - serosanguinal choroidal effusion in one eye (16 months/range 5–32)
Meng et al. 2014 [11]	Ciliary block glaucoma	Eleven eyes (of nine patients)	- Trabeculectomy with peripheral iridectomy	Primary angle closure glaucoma	3.9 ± 4.1 (range 1–11) days	Lens disproportion and lens–ciliary body apposition in small eyes and anterior hyaloid changes, with increased hydraulic resistance, are thought to be the major pathophysiological factors. In addition, unfavorable vitreous flow and an expansion of the choroidal volume in small eyes may be involved in malignant glaucoma. These anatomical characteristics lead to aqueous humor misdirection into the vitreous cavity and cause forward movement of the lens–iris diaphragm	AC infusion followed by single port 25-G anterior vitrectomy, phaco + IOL in phakic eyes, posterior capsulectomy with anterior hyaloid face removal until sudden pupil dilatation and deepening of the AC is achieved	Although IOP control was achieved in all eyes after surgery, two eyes required long-term topical antiglaucoma medication (6–18 months)
Rekas et al. 2015 [12]	Malignant glaucoma	Twenty-two eyes (22/1689 glaucoma surgeries, 2.3% penetrating glaucoma procedures, 0% of non-penetrating procedure)	- Phacoiridencleisis (40.9%) - Phacotrabeculectomy (22.7%) - Iridencleisis (18.2%) - Trabeculectomy (13.6%) - Seton valve implantation (4.6%)	Penetrating glaucoma surgery	Average 61 days (1–840 days)	Aqueous humor accumulates in the area of the vitreous cavity due to ciliary block, and, as a result of this, there is an increase in the vitreous pressure that is transferred to the structures of the anterior segment causing a forward movement of the lens-iris diaphragm	25-gauge vitrectomy with iridectomy/iris-lens/iridocorneal/iris-capsule adhesions released. Authors suggest quick implementation of surgical treatment.	IOP ≤ 21 mmHg without topical hypotensive agents in 49.0% after 12 months, IOP ≤ 21 mmHg with a maximum of two topical hipotensive agents in 85.7% after 12 months (mean 405 days / range 7–1440 days)
Premsenthil et al. 2012 [13]	Positive vitreous pressure glaucoma	One eye	LPI	N/A	1 year	In malignant glaucoma, a vicious cycle of poorer vitreous fluid conductivity and increased transvitreal pressure is established. This results in compression of the vitreous gel, progressive forward displacement of the lens-iris diaphragm and eventual direct closure of the AC angle despite the presence of patent iridotomy	PPV, phacoemulsification, primary posterior capsulotomy, and posterior chamber IOL implantation.	IOP stabilization (6 months)
Pasaoglu et al. 2012 [14]	Malignant glaucoma	Two eyes	- Trabeculectomy (one pseudophakic eye) - Phaco + IOL, recurrence of malignant glaucoma 9 months after PPV with capsulotomy and peripheral iridectomy (one eye)	N/A	1 week–1 month	Relationship of the lens, ciliary body, and anterior hyaloid face causing an aqueous misdirection and blockade was suggested in the pathogenesis. The aqueous has been thought to be entrapped inside the vitreous cavity as aqueous pockets resulting in forward movement of the iris-lens diaphragm which causes the secondary angle closure glaucoma.	Patients were treated successfully by using an AC approach consisting of a capsulo-hyaloidectomy and anterior vitrectomy performed through a peripheral iridectomy, creating a permanent passage between the AC and vitreous cavity by eliminating the aqueous misdirection.	IOP stabilization (5 months)
Zarnowski et al. 2014 [15]		Ten eyes	- Phacotrabeculectomy (four eyes) - Trabeculectomy (three eyes) - phaco + IOL (three eyes)	- History of primary angle closure - Hyperopia	N/A	All of the tissues (iris, lens capsule, zonule, and anterior vitreous) have to be removed to create a permanent passage between the AC and the vitreous cavity. Part of the problem is that, during conventional vitrectomy peripheral vitreous can hardly be completely removed, and that is why relapse rate may be very high.	Anterior hyaloidectomy combined with peripheral iridectomy, zonulectomy, and peripheral capsulectomy	IOP stabilization (12 months/range 6–18.0 months)
Shahid et al. 2012 [16]	Direct lens block glaucoma	(0.4–6% of incisional surgery for primary angle-closure glaucoma)	- Incisional surgery for primary angle-closure glaucoma - After cataract extraction - Spontaneously, LPI, trabeculectomy scleral flap suture lysis, cyclophotocoagulation, use of miotics and trabeculectomy bleb needling. Sporadically in association with infection, retinopathy of prematurity, retinal detachment, retinal vein occlusion and trauma.	- Tendency in an individual (as it often occurs in fellow eyes)	Immediate postoperative period to many years after surgery	- Posterior diversion of aqueous flow causes accumulation of aqueous behind a posterior vitreous detachment with secondary forward movement of the iris-lens diaphragm - Laxity of lens zonules coupled with pressure from the vitreous leads to forward lens movement. A vicious circle is set up in that the higher the pressure in the posterior segment, the more firmly the lens is held forward. - Choroidal expansion	Cycloplegics, aqueous suppressants, osmotic agents and steroids. Cataract extraction with IOL implantation, posterior capsulotomy and vitrectomy in phakic eyes. In aphakic/pseudophakic eyes the following procedures were performed: - Nd:YAG laser capsulotomy and disruption of anterior hyaloid face - Transscleral cyclodiode laser - Vitrectomy.	N/A
Madgula et al. 2014 [17]		Ten eyes	- Phacotrabeculectomy - Trabeculectomy with mitomycin - Phaco + IOL - ECCE - Phacovitrectomy	N/A	N/A	Various anomalies of the ciliary body, choroid, lens, zonule, and vitreous which may lead to posterior diversion of aqueous humor into the vitreous cavity have been proposed. Anterior rotation of the ciliary body processes can lead to cilio-lenticular touch and ciliary block	Zonulo-hyaloido-vitrectomy (anterior approach)	Malignant glaucoma recurred in four eyes. The reasons are blockage of the channel by vitreous or fibrin. Complications: - macular hole - cystoid macular edema - corneal decompensation (50.2 ± 27.2 months)
Lois et al. 2001 [18]	Malignant glaucoma	Five pseudophakic eyes	- Phacotrabeculectomy (three eyes) - Phaco + IOL (one eye) - Trabeculectomy (one eye)	N/A	N/A	Existence of an abnormal anatomic relationship between the ciliary processes, the crystalline lens, or IOL and anterior vitreous face, which leads to misdirection of aqueous fluid into the vitreous cavity	Zonulo-hyaloido-vitrectomy (anterior approach)	Resolution of the malignant glaucoma was achieved in all cases. In one patient with extensive anterior synechiae, bleb failure occurred after the resolution of the malignant glaucoma. This patient was treated successfully with a guarded filtration procedure supplemented with 5-fluorouracil. (1–9 months)
Varma et al. 2014 [19]	Malignant glaucoma	Twenty eyes	Phaco + IOL	- Female hyperopic patients	5.8 ± 7.1 weeks	Ciliolenticular block presumably induced by anterior movement of the lens–iris diaphragm, poor vitreous conductivity, and choroidal expansion.	- Medical therapy: cycloplegics and aqueous suppressants - Nd:YAG Iridozonulohyaloidotomy - AC reformation/IOL pushback - Iridozonulohyaloidovitrectomy (pars plana approach)	Symptoms resolved in (IOP control with topical medications): Medical therapy (two eyes) Iridozonulohyaloidotomy (seven eyes) AC reformation–IOL pushback (six eyes) Iridozonulohyaloidovitrectomy (five eyes)
Dave et al. 2013 [20]	Malignant glaucoma	Twenty-eight eyes	Trabeculectomy (11 eyes) Cataract surgery (ten eyes) Combined cataract and glaucoma surgery (seven eyes).	N/A	N/A	Misdirection of aqueous into or behind the vitreous body that is responsible for an increase in vitreous volume and subsequent obliteration of the anterior and posterior chambers.	1. Medical management included topical cycloplegics (atropine or cyclopentolate) and topical / oral aqueous suppressants. Intravenous 20% mannitol in a dose of 1 mg/kg was administered at the discretion of the treating physician. 2. Nd:YAG Laser hyaloidotomy in pseudophakic eyes through an existing peripheral iridotomy or beyond the haptic of the IOL. 3. Vitrectomy-hyaloidotomy-iridotomy (pars plana approach) 4. Transscleral cyclophotocoagulation	IOP <21 mmHg with up to two topical medications in: 1) four eyes 2) seven eyes 3) four eyes 4) 11 eyes In two eyes repeated transscleral cyclophotocoagulation was required — in one eye symptoms have not resolved (192 days/ range 35–425 days)
Stumpf et al. 2008 [21]	Aqueous misdirection syndrome	Five eyes	- Phacoemulsification (one eye) - Phacoemulsification and trabeculectomy (three eyes) - Phacotrabeculectomy (one eye)	- Hyperopia / short axial length (< 22.64 mm) - Narrow iridocorneal angle - Shallow AC	Directly after surgery to 2 years	n/a	Large posterior Nd:YAG laser capsulotomy / LPI was ineffective in deepening the AC. Transscleral cyclophtotocoagulation (9–33 pulses) of 2–2.5 W for 2 s in 1–2 quadrants	All cases responded rapidly, though in one eye required a subsequent second application a year later (15–96 months)
Heindl et al. 2013 [22]		One eye	Phaco + IOL	N/A	10 days	Ultrasound biomicroscopy shows anterior rotation of the ciliary body processes suggests a blocking mechanism between ciliary processes, IOL, lens capsule, and anterior vitreous face.	23-gauge PPV combined with iridectomy and peripheral removal of lens capsule behind the iridectomy site at the 1–2-o’clock position.	Complete resolution (6 months)
Prata et al. 2013 [23]	Malignant glaucoma	Thirty-one eyes	- Trabeculectomy with mitomycin (14 eyes) - Phaco + IOL (8 eyes) - Phacotrabeculectomy with mitomycin (three eyes)	- Plateau iris configuration	Median 29 days (range 2–364 days).	Anteriorly rotated ciliary in ultrasound biomicroscopy. A plateau iris configuration, defined as large and/or anteriorly positioned ciliary body abutting the peripheral iris, partial visibility or absence of the ciliary sulcus, an iris root that angulates forward and then centrally, presence of a central flat iris plane, and irido-angle contact was noticed in 85% of eyes	N/A	N/A
Debrouwere et al. 2012 [24]	Malignant glaucoma	Twenty-four eyes	- Trabeculectomy (11 eyes) - Phaco + IOL (eight eyes) - Phacotrabeculectomy (one eye) - Diode laser cyclodestruction (one eye) - ICCE (one eye) - Iridotomy (one eye) - Nd:YAG LPI (one eye)	- Hyperopia - Chronic angle-closure glaucoma - Shallow AC after surgery	N/A	Anterior rotation of the ciliary body, so the aqueous produced by the ciliary body cannot follow its normal pathway and accumulates behind the iris–lens diaphragm. Subsequent increased pressure in the posterior segment results in an anterior displacement of the iris–lens diaphragm, AC flattening and secondary angle	1) Medical treatment 2) Nd:YAG laser capsulotomy + hyaloidotomy 3) Vitrectomy 4) Anterior vitrectomy + iridectomy–zonulectomy 5) Full vitrectomy–phaco–iridectomy–zonulectomy	Relapse in: 1) 100% eyes 2) 75% eyes 3) 75% eyes 4) 66% eyes 5) 0% eyes (61 days/range 13–228 days)
Arya et al. 2004 [25]	Malignant glaucoma	One eye	Nd:YAG laser posterior capsulotomy in pseudophakic eye	- Alteration in the anatomic relationship involving the ciliary body, anterior hyaloid membrane face, and vitreous	Four days	B-scan ultrasonography showed aqueous pockets in the vitreous. Persistent liquefaction of the vitreous leading to shifting of vitreous gel forward and misdirection of aqueous posteriorly.	Nd:YAG LPI, medical treatment followed by PPV with air administration to the AC	Recurrence — 8 days after PPV. Repeated PPV stabilized IOP (6 months)
Brooks et al. 1989 [26]	Malignant glaucoma	One eye	NdYAG LPI		Five days	Strong tropine-like mydriatics and strong miotics in such patients with very shallow ACs and an unstable lens iris diaphragm.	Panretinal photocoagulation. Medical treatment	Resolution after cancelation of mydriatics
Wu et al. 2016 [8]	Malignant glaucoma	Thirty-eight eyes (of 1432 eyes undergoing antiglaucoma surgical treatment for primary angle-closure glaucoma)	- After trabeculectomy (32 eyes — 84.2%) - During trabeculectomy (four eyes — 10.5%) - After combined glaucoma/cataract surgery (two eyes — 5.3%)		During or after surgery	1) Ciliolenticular block or anterior hyaloid obstruction 2) Slackness of lens zonules 3) Severe postoperative inflammation 4) Non ciliary block. It was observed that those patients who developed malignant glaucoma during surgery all showed a high stress level and poor operative cooperation. We speculated that such status caused constriction of extraocular muscles pressing forward, bringing about the increase of ocular venous pressure.	All patients were initially given medical treatment (four eyes). If medical treatment failed: 1) laser posterior capsulotomy with hyaloidotomy (two pseudophakic eyes) Phakic eyes underwent surgical treatment 2) anterior vitrectomy-reformation of AC was chosen if IOP increased non-progressively, the lens is transparent and the ACdepth is on grade II or shallower according to Spaeth (13 eyes) 3) phaco + IOL with goniosynechialysis and reformation of AC was indicated if progressive IOP increase is associated with the development or worsening of cataract. Anterior vitrectomy procedure was performed if AC failed to form during the surgery (ten eyes) 4) vitreous aspiration or anterior vitrectomy was performed if patients developed malignant glaucoma during surgery (nine eyes). In refractory relapsed eyes with low visual potential, transscleral cyclophotocoagulation was performed.	Thirty of 38 eyes did not require topical treatment achieving IOP <21 mmHg. With topical hipotensive agents: IOP <21 mmHg in four eyes, IOP ranged 23–26 mmHg in four eyes. Complications: One eye exhibited bleeding at the entry site of vitrectomy into vitreous cavity. Corneal endothelial decompensation occurred to one eye, and another eye showed positive response to medical therapy but allergy to atropine. (27.1 ± 9.1 months)
Lazar et al. 1981 [27]	Anterior pupillary block	Two eyes	- Surgical peripheral iridectomy (one eye) - Penetrating keratoplasty (one eye)		8 h–3 days	The mechanism is caused by adhesions between the iris and hyaloid membrane/lens. Pressure disparity between posterior and AC. leads to backward movement of the lens. Further interference of aqueous flow due to by iridocorneal adhesion.	Aspiration of fluid from the posterior chamber with a 25-gauge needle in the slit lamp — under slit-lamp visualization — through cornea and iris/iridectomy. Medical: 1% atropine, 10% phenylephrine, acetazolamide + hypertonic agents (one patient)	A complete reformation of the AC occurred immediately in one eye or in 30 min in one eye
Ozeki et al. 2010 [28]	Ciliovitreal block	One eye	Trabeculectomy with unplanned zonulectomy		Two days	Ciliovitreal block caused by the vitreous herniation through the peripheral iridectomy to the limbal incision with flat bleb and AC	Medical: topical atropine, beta-blocker, oral acetazolamide, and intravenous mannitol. Healon5 injected through a paracentesis	This approach was successful, and the malignant mechanism did not recur over a period of almost 2 weeks, until a more rigid and deep AC was constructed by cataract surgery.
Massicotte et al. 1999 [29]	Pseudomalignant glaucoma	Two eyes	Partial vitrectomy with C_3_F_8_ tamponade (leaving the anterior hyaloid) in phakic eyes		1–6 days	Obstruction of aqueous flow, either by residual anterior hyaloid or by fibrin and other inflammatory debris at the level of the ciliary body–zonular apparatus. Anterior rotation of the ciliary processes (arrowhead) and an axially shallow and fibrin-filled AC was found in ultrasound biomicroscopy	1) Medical 2) Nd:YAG LPI and hyaloidectomy through the zonules was performed and resulted in only transient deepening of the AC 3) Vitreous chamber gas was aspirated, and tissue plasminogen activator 10 mg was given intracamerally 4) Phacoemulsification, air–fluid exchange with removal of C_3_F_8_ gas and peripheral iridectomy from a posterior approach through the remaining vitreous, zonules, and iris (one eye)	IOP stabilization and AC reformation (1 year)
Francis and Babel 2000 [30]	Malignant glaucoma	One eye	PPV with lensectomy, scleral buckling and vitreous injection of 18% SF_6_		Fourteen days	Aqueous misdirection apparently requires an intact hyaloid, perhaps with decreased permeability to aqueous or reduced surface area for fluid transfer. Hydration of the vitreous and increase in vitreous volume, exacerbated by expansile gases, may result in elevated IOP and axial shallowing of the AC, even in the presence of a patent iridectomy.	Surgical peripheral iridectomy without any effect on the AC. PPV with a minor buckle release	AC deepening and IOP normalization
Al Bin Ali et al. 2016 [31]	Aqueous misdirection syndrome	Sixty-nine eyes	- Cataract surgery (17 eyes — 25%) - Trabeculectomy (nine eyes — 13%) - Phacotrabeculectomy (43 eyes — 62%)		n/a	The authors believe that removal of the anterior hyaloid face is the key to resolving the symptoms of fluid misdirection syndrome, rather than debulking the vitreous. Though they suggest that a two-port PPV with surgical microscope lighting can be as effective as a 3-port procedure	Two-port PPV or three-port PPV. In pseudophakic eyes, a hyaloido-zonulo-iridectomy and posterior capsultomy was performed	Primary functional success rate of PPV in reducing IOP to <21 mmHg was 81%, with recurrence rate 11%. Intraoperative and postoperative complications included retinal detachment in two eyes and endophthalmitis in one eye (3–156 months)
Bitrian and Caprioli 2010 [32]	Aqueous misdirection syndrome	Five eyes	- Phacoemulsification (one eye) - Phacoemulsification, trabeculectomy, laser suture lysis (one eye) - Ahmed implant, phacoemulsification, Ahmed revision (one eye) - Phacoemulsification, Nd:YAG laser capsulotomy (one eye) - Phacoemulsification, Ahmed implant, Nd:YAG laser capsulotomy (one eye)		One day–8 months	The exact pathophysiology is not understood fully, but diverse anomalies of the ciliary body, choroid, lens, zonule, and vitreous have been suggested, causing a posterior diversion of the aqueous humor into the vitreous cavity. An anterior rotation of the ciliary body processes, leading to ciliolenticular touch and ciliary block, has been suggested.	Anterior vitrectomy with hyaloido-zonulectomy, peripheral iridectomy (pseudophakic eyes). The infusion line was placed in the AC to deepen it.	This surgical procedure was successful in resolving the aqueous misdirection in all eyes. An anatomic success has been achieved, in most cases IOP normalized (1–13 months)
Tsai et al. 1997 [33]	Malignant glaucoma / ciliary block	Twenty-five eyes	Predominantly trabeculectomy. Also cataract extraction, surgical peripheral iridectomy, Molteno implant		n/a	Anatomical obstruction of aqueous flow at the anterior hyaloid/zonule-lens/ciliary process interface. The authors conclude that surgical vitrectomy in the presence of an intact posterior capsule may preclude the surgical resolution of aqueous misdirection.	Anterior vitrectomy or PPV. Additional concomitant cataract extraction should be performed in phakic eyes, preferably with posterior capsulotomy.	Various outcomes depending on the performed surgical procedure. In eyes with pre-existing cataract, combined lens extraction, primary posterior capsulectomy and surgical vitrectomy should be performed (10 days–65 months)
Greenfield et al. 1999 [34]	Aqueous misdirection	Ten eyes	Baerveldt glaucoma drainage device implantation. In one eye, concurrent trabeculectomy.		1–343 days	Overfiltration associated with the drainage device, which developed within 2 months of the implantation We propose that this syndrome results from a cascade of events, which is initiated by external ligature release. This produces immediate overfiltration and shallowing of the AC, anterior rotation of the lens–iris diaphragm, and posterior diversion of aqueous.	Nd:Yag hyaloidotomy. PPV alone (pseudophakic eyes) or with lensectomy (phakic eye)	Normalization of anterior segment anatomy was achieved with aqueous suppression and cycloplegia in one eye; in four eyes after Nd:YAG capsulotomy; in two eyes after PPV; in one eye after PPV with lensectomy; two eyes after PPV with IOL explantation (1–23 months).
Lynch et al. 1986 [35]	Malignant glaucoma, ciliovitreal block	Four eyes	- ECCE + IOL and iridectomy (one eyes) - ECCE + IOL and trabeculectomy (one eye) - ICCE with AC IOL implantation and iridectomy (one eye)	- Small eyes with anatomically narrow AC / short axial length - History of narrow angle glaucoma	2–7 days	Increased resistance of the anterior vitreous is thought to initiate or perpetuate the posterior flow of aqueous humor. This resistance might occur at the level of the ciliary body, and has been called ciliovitreal block. Pseudophakic eyes, especially after ECCE often contain residual cortical lens material, which incites a cellular and fibrous reaction in the equatorial region of the lens capsule. This may contribute to blockage of the aqueous flow through the lens zonules or between the ciliary processes and capsule.	Nd:YAG hyaloidotomy (one eye — unsuccessful) PPV with excision of the lens capsule and zonules (four eyes)	IOP stabilization with or without topical medications and AC deepening in three of four eyes
Tomey et al. 1987 [36]	Malignant glaucoma, ciliary block, aqueous misdirection	four eyes	- ECCE + IOL (one eye) - ECCE + IOL with trabeculectomy (three eyes)		1–10 days	Postoperative wound leakage may be a causative factor. It seems plausible that the initial forward movement of the iris-lens diaphragm caused by the wound leak (and perhaps also aggravated by the absence of iridectomy in some cases) starts a cycle of aqueous misdirection and subsequent accumulation in the posterior segment. Interestingly, trabeculectomy openings may very well be considered deliberate leaks.	Nd:YAG hyaloidotomy as first step management PPV (two eyes)	In two eyes IOP was stabilized and AC deepening was achieved with Nd:YAG hyaloidotomy. In two eyes, PPV was required; in one eye it was redone with IOL removal (6–18 months)
Harbor et al. 1996 [37]	Malignant glaucoma	Twenty-four eyes	- Trabeculectomy (six eyes) - Trabeculectomy with mitomycin (three eyes) - Nd:YAG LPI (four eyes) - Central retinal vein occlusion (one eye) - ECCE + IOL (six eyes) - ECCE + IOL with trabeculectomy (four eye)		1–120 days	Posterior misdirection of aqueous flow into or behind the vitreous body, with subsequent increase in vitreous volume and obliteration of the anterior and posterior chambers.	Vitrectomy without lensectomy (nine eyes) Vitrectomy with lensectomy (six eyes) Nd:YAG LPI (two eyes) Nd:YAG laser posterior capsulotomy (three eyes) ECCE (one eye) Surgical peripheral iridectomy (one eye) AC reformation (one eye) Argon laser gonioplasty (one eye)	In phakic eyes, the initial PPV was successful in seven of svene eyes that underwent lensectomy, and in five of seven eyes that did not undergo lensectomy. In pseudophakic eyes the initial vitrectomy was successful in 9/10 eyes. Perioperative complications included: - transient serous choroidal detachment in two eyes - transient exudative retinal detachment in one eye - suprachoroidal hemorrhage in one eye (1–89 months)

Abbreviations used: AC, anterior chamber; ECCE + IOL, extracapsular cataract extraction with intraocular lens implantation; ICCE, intracapsular cataract extraction; IOL, intraocular lens; IOP, intraocular pressure; LPI, laser peripheral iridotomy; Nd:YAG, neodymium:yttrium–aluminum–garnet; phaco + IOL, phacoemulsification with intraocular lens implantation; PPV, pars plana vitrectomy

**Table 3:** Common risk factors for *fluid misdirection syndrome*

Onset of fluid misdirection	Risk factor	Influence on pathophysiology
During phacoemulsification with IOL implantation or trabeculectomy	- Higher levels of anterior chamber irrigation [3]	Residual cortical fiber irrigation maneuver (when residual cortical fibers are being removed, the narrow stream of balanced salt solution generated by the narrow hydrodissection cannula tip may distort the posterior capsule locally) resulting in localized anterior displacement of the contiguous posterior capsule, creating a subcapsular or vitreal space through which the balanced salt solution may move via the zonular fibers.
	- Coughing [6] or high stress level with poor intraoperative cooperation [8]	Increased localized pressure in the eye. Constriction of extraocular muscles pressing forward, bringing about the increase of ocular venous pressure.
	- Hyperopia [3, 6]	Small hyperopic eyes with shallow anterior chamber, leading to a decrease of surgical space.
	- Intumescent cataract [4, 6]	Posterior capsule flaccidity might result in bulging or billowing forward
	- Pseudoexfoliation [4]	Lax zonular fibers might facilitate the fluid passage through the region of the zonular fibers.
During penetrating keratoplasty with extracapsular cataract extraction and IOL implantation	- Open sky irrigation/aspiration [5])	Irrigation under the iris, anteriorly to the anterior capsule
Following phacoemulsification with IOL implantation, trabeculectomy or phacotrabeculectomy	- Hyperopia [10, 19, 21]	Abnormal anatomic relationship between the ciliary body, anterior hyaloid, and lens in hyperopic eyes. Anterior movement of the lens–iris diaphragm accompanied with poor vitreous conductivity and choroidal expansion.
	- History of angle closure [11, 15, 24, 35]	Increased resistance of the anterior vitreous initiates the posterior flow of aqueous humor.
	- Plateau iris configuration [23]	Anterior rotation of the ciliary body, so the aqueous produced by the ciliary body cannot follow its normal pathway and accumulates behind the iris–lens diaphragm.
	- Shallow anterior chamber after surgery [21]	A forward displacement of the anterior vitreous with apposition of the anterior hyaloid face against the lens and ciliary body prevents the normal anterior flow of aqueous humor.

Abbreviations used: IOL, intraocular lens

## Results

We collected and analyzed 55 articles dated from 1951 to 2016. Most of the articles related to intraoperative complications were published since 2010, though the first description by MacKool is from 1993 [4]. As of now, about 20 cases of this syndrome have been described, commonly during uneventful phacoemulsification. The most numerous group of cases was collected by Lau et al. [3] Six eyes were described, which was 1.45% cases among all phacoemulsification procedures (Table 1).

Articles referring to malignant glaucoma are definitely more numerous, and over 200 cases have been described worldwide. The most abundant was a study described by Al Bin Ali et al. [31] which referred to 69 eyes. Recent studies focus on the optimal surgical approach, as the outcome is still not satisfactory (Table 2).

Common risk factors for *fluid misdirection syndrome* have been summarized in Table 3.

## Discussion

### Mechanisms of acute fluid misdirection syndrome

The *fluid misdirection syndrome* is a rare clinical condition characterized by an axially very shallow anterior chamber with the absence of suprachoroidal effusion or hemorrhage and no noticeable pathology of the iris–lens diaphragm. It usually occurs during uneventful phacoemulsification particularly in hyperopic eyes [3]. It is probably underreported; anecdotally, many surgeons admit having experienced it occasionally. The common theme is that it manifests toward the end of irrigation/aspiration (I/A), making the completion of I/A or the insertion of an intraocular lens impossible because of flat anterior chamber. The accumulation of fluid in the posterior segment engenders increase in posterior pressure, resulting in anterior displacement of the iris–lens diaphragm, axial and peripheral anterior chamber flattening, and secondary angle closure. The severity of this condition was underlined by using the name *acute intraoperative rock-hard eye syndrome*.

The integrity of the posterior chamber (PC)–anterior hyaloid membrane (AHM) barrier during phacoemulsification has been thoroughly evaluated ex vivo through contrast-enhanced magnetic resonance imaging and in the Miyake–Apple view on porcine eyes [38, 39]. Prolonged irrigation and deflation/inflation of the anterior chamber are risk factors of AHM detachment, while hydrodissection is associated with an AHM tear [38]. Furthermore, ocular viscosurgical devices (OVD) with higher molecular weight or higher concentration of sodium hyaluronate predisposed the eye to an increased risk of PC-AHM impairment during hydrodissection [39].

In vivo, breaking the PC–AHM barrier is extricated by the residual cortical fiber irrigation maneuver. It is excessive irrigation which forces fluids into the PC, thus, the term of *infusion misdirection syndrome* or *intraoperative fluid misdirection* has been suggested [4, 6]. The narrow flow is usually generated by a 27G straight or curved hydrodissection cannula tip. However, it might take place during I/A with a typical irrigation probe, or phacoemulsification at the time of removing the last remaining nuclear pieces. It might be noticeable that the posterior capsule is flaccid — bulging or billowing forward [4]. Lau et al. [3] claim that higher levels of anterior chamber irrigation might be a risk factor.

Miscellaneous routes for balanced salt solution entering the anterior vitreous or Berger’s space have been described. Obviously, a radial extension of capsulorhexis might enable direct access of fluid into the retrocapsular space [4]. In these mild cases, administration of OVD into the anterior chamber might be possible, as well as dry aspiration of retrocapsular fluid [5]. Presumably, if irrigation is performed anteriorly to the anterior capsular flap, fluid could more easily make its way through the zonules.

In intact capsules, the zonular dehiscence may enable fluid to flow in an unusual pattern, facilitating its entrapment in the PC. This elucidates why this syndrome is commonly associated with lax zonular fibers, i.e., in exfoliation, dense/brunescent cataracts, or spherophakia. However, it can develop in the absence of any clinically detectable zonular fiber weakness or disruption [4], and might be associated with spontaneous PC/vitreous pressure elevation induced by intraoperative coughing [6]. As habitually shallowing of the anterior chamber is irreversible, Olson et al. defined the term *subcapsular fluid entrapment*.

### Optimal treatment for acute fluid misdirection syndrome

Faced with these situations, pars plana decompression is required. Exceptionally spontaneous posterior capsule rupture and liberation of the entrapped fluid has been described [5]. Prior to performing a posterior decompression, the surgeon must be definitely certain that there is no evidence of choroidal effusion or hemorrhage. The decompression might be done by puncture with a straight needle 3 mm from the rim and then aspiration of retrolenticular fluid [3–5]. Vitreous traction might be a concern when performing this procedure. Furthermore, the treatment has not always been described as successful [5].

Hence it would be preferable to use a small-gauge trocar/cannula vitrectomy cutter (23-, 25-, or 27-gauge) [7]. The incision in the pars plana should be made after displacing the conjunctiva and then fashioning a beveled incision, as is modern practice for pars plana entries. The cutter can then remove retrocapsular fluid using a high cut rate.

### Mechanisms of chronic fluid misdirection syndrome

**Fig. 1:**
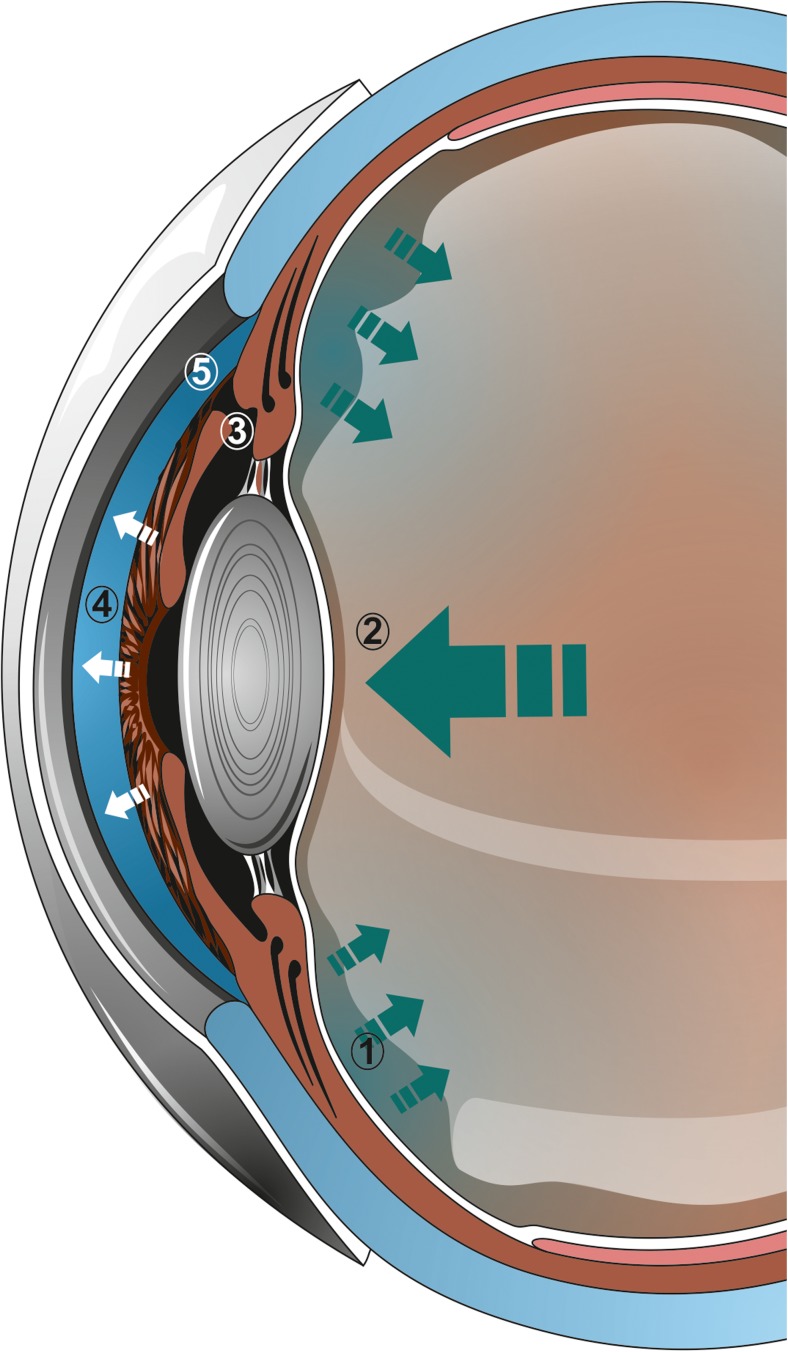
Mechanism of *chronic fluid misdirection syndrome*. The aqueous humor secreted by the ciliary epithelium is not directed to the anterior chamber (*1*), which leads to positive vitreous pressure (*2*). This occurs despite the existence of a patent iridotomy or iridectomy (*3*). Subsequently a diffusely flat anterior chamber (*4*) and angle closure (*5*) can be observed with precipitous rise in intraocular pressure

This syndrome has also been described as occurring from hours to months, or years, after the initial surgery [9]. *Malignant glaucoma is* a recalcitrant and potentially devastating secondary angle-closure glaucoma. In 1869, von Graefe described a rare postoperative complication characterized by flattening of the anterior chamber and elevated intraocular pressure (IOP). As a result of its poor response to conventional treatment, it was called *malignant glaucoma* [1]. The term was further justified by the devastating effect of using pilocarpine as an attempted treatment for this condition. It is recognized to comprise the diagnostic triad of a diffusely flat anterior chamber, high intraocular pressure, and aqueous pooling that is sometimes visible in or in front of the anterior vitreous. This occurs despite the existence of a patent iridotomy or iridectomy (Fig. 1). It is best known following trabeculectomy, but has been reported following a wide variety of anterior segment procedures, including cataract extraction or implantation of several glaucoma drainage devices, i.e., Ahmed, Molteno, Baerveldt [32–34]. Furthermore, it can occur subsequent to laser peripheral iridotomy, surgical peripheral iridectomy, capsulotomy, cyclophotocoagulation, phacotrabeculectomy, trabeculectomy scleral flap suture lysis, trabeculectomy bleb needling, or initiation of topical miotic [5, 10, 12, 24, 40]. Although pars plana vitrectomy is an effective treatment for *malignant glaucoma*, it does not rule out the development of this syndrome postoperatively [29, 30]. Furthermore cases of *malignant glaucoma* have been described following vitrectomy, particularly in phakic eyes [29, 30, 41]. In a study conducted by Matlach et al., the IOP in *malignant glaucoma* following trabeculectomy was slightly lower than after other procedures; furthermore, this group of patients required fewer topical IOP-lowering medications [42].

There is an anatomical predisposition for *malignant glaucoma*. Most patients have chronic angle-closure glaucoma or an anatomically narrow filtration angle. In other studies, plateau iris configuration and hyperopia have been defined as risk factors [10, 15, 23, 24]. Additionally, anterior rotation of the ciliary body processes in ultrasound biomicroscopy might be significant for averting aqueous fluid flow [22]. Malignant glaucoma is indeed more common in women than in men. In women, the location of the lens is more forward than that of the lens in men, resulting in not only a 4% shallower anterior chamber but also a narrower space between the lens equator and ciliary body. Therefore, women are more prone to developing a misdirection of the aqueous flow [43]. Chandler et al. proposed that laxity of lens zonules coupled with pressure from the vitreous leads to forward lens movement [44]. In spherophakia, zonular dehiscence might be accompanied by increased lens thickness, hence facilitating anterior chamber shallowing [45].

Due to the fact that the aqueous humor secreted by the ciliary epithelium is not directed to the anterior chamber, the term *aqueous misdirection syndrome* has been proposed [10]. It might accumulate in the vitreous or adjacent to it in Berger’s space. A forward displacement of the anterior vitreous with apposition of the anterior hyaloid face against the lens and ciliary body might be significant in averting the flow of aqueous humor [9]. Furthermore, the aqueous might become entrapped inside the vitreous cavity as “aqueous pockets” [14, 40]. Other studies suggest that anterior vitreous and anterior hyaloid face condensation result in reduced permeability to the aqueous. With that, vitreous detachment would facilitate trapping fluid in the posterior segment [10]. Little et al. [9] suggest that aqueous might accumulate in front of the anterior vitreous, and its pooling can be sometimes visible.

The misdirection of aqueous leads to increasing the pressure of the posterior chamber vitreous (*positive vitreous pressure glaucoma)* [13] and anterior displacement of the lens–iris diaphragm. Consequently, a characteristic diffuse shallowing of the anterior chamber can be observed, with subsequent angle closure and rise in intraocular pressure [10]. The aqueous misdirection is inevitable — Shaffer and Hoskins postulated a valve-like mechanism by which aqueous humor was “misdirected” posteriorly [46]. A vicious circle is set up, in that the higher the pressure in the posterior segment, the more firmly the lens is held forward [13, 16].

Quigley et al. suggest that rather than a one-way ball valve mechanism, choroidal expansion could play a significant role in malignant glaucoma. In an average human eye, the vitreous volume is approximately 5000 μl and the choroidal volume is about 480 μl, while the anterior chamber volume is 150 μl [47]. From solid geometry and ocular dimensions, choroidal expansion by 20% occupies a volume 100 μl, equal to the volume of the anterior chamber [48]. A 100-μm uniform choroidal expansion could increase IOP to 60 mmHg [47]. With the expansion of the choroid, the absolute pressure in each compartment of the eye increases. This induces a volume loss from the anterior chamber, with increased aqueous outflow. As the pressure in the posterior globe is higher than in the anterior chamber, an anterior movement of the vitreous, carrying the lens and iris with it, leads to further anterior chamber narrowing. This additionally decreases the outflow, causing a vicious circle.

### Treatment strategies for chronic fluid misdirection syndrome

Malignant glaucoma would be more likely to occur in eyes with higher than normal resistance to vitreous fluid flow. Normal vitreous does not limit the free passage of water, and its fluid conductivity decreases under an increased pressure differential [49]. If the transvitreous flow is limited and insufficient to equalize the difference in pressure between the vitreous chamber and anterior chamber, the vitreous compresses more. This further decreases its fluid conductivity. The surface through which aqueous exits the vitreous is limited by the ciliary body at its perimeter and apposition of vitreous to the lens centrally, forming a doughnut-shaped zone. The compression of vitreous and its anterior movement gradually limits the diffusional area. Furthermore in hyperopic eyes with smaller axial length and relatively larger lens the doughnut-shaped zone would be only half as large as normal-sized eyes [48].

Tomey et al. [36] claim, that postoperative wound leakage following cataract surgery may be a causative factor for malignant glaucoma. It seems probable that the initial forward movement of the iris–lens diaphragm caused by the wound leak starts a cycle of aqueous misdirection and subsequent accumulation in the posterior segment. This might be aggravated by the absence of iridectomy in some cases. Interestingly, filtration surgery with increased aqueous outflow might also be considered as a triggering factor.

The treatment strategies for malignant glaucoma are typically aimed at managing IOP and restoring normal anterior segment anatomy. Medical therapy is reported to be successful. Cycloplegics draw the lens–iris complex posteriorly, widen the ciliary body diameter, increasing forward diffusional area for fluid to leave the posterior vitreous cavity. Osmotic agents remove fluid from the eye, and aqueous suppressants decrease its production. Although medical treatment can help partially or completely stabilize malignant glaucoma, they do not address the underlying pressure imbalance; thus the relapse rate is described to be as high as 100% [24].

In aphakic and pseudophakic eyes neodymium:yttrium–aluminum–garnet (Nd:YAG) laser iridotomy with anterior hyaloidotomy and posterior capsulotomy (all through the same location) might stabilize the intraocular pressure [9]. This approach leads to relieving aqueous that might be entrapped within the vitreous. Ultrasound biomicroscopic imaging revealed that anterior rotation of the ciliary body and anterior chamber shallowing normalize after rupture of the anterior hyaloid face [50]. However, the procedure might have a short-term effect with a high recurrence rate of 75%, presumably because the primary mechanism of misdirection is not counteracted, allowing new aqueous to accumulate in the vitreous cavity [24]. Furthermore, there might be an inflammatory reaction in blocking the flow of aqueous across the zonules or between the lens capsule and ciliary processes, especially in eyes with residual cortical lens material [35]. Some authors underline the efficacy of transscleral cyclophotocoagulation [20, 21]. The coagulative necrosis and shrinkage of the ciliary processes disrupts the ciliary–hyaloid interface. In addition to decreasing aqueous production, this disruption may subsequently allow normal aqueous flow and mechanical posterior rotation of the ciliary body. This method needs to be considered, although some authors prefer to use a non-destructive intervention, especially in a patients with well-seeing eyes [20]. Importantly, this approach does not complicate a subsequent surgical procedure [21]. It is suggested that although cyclophotocoagulation helps to achieve resolution in most eyes, performing vitrectomy with hyaloidotomy and iridectomy with implantation of a glaucoma drainage device in eyes in which laser hyaloidotomy failed could be a better option [20].

**Fig. 2:**
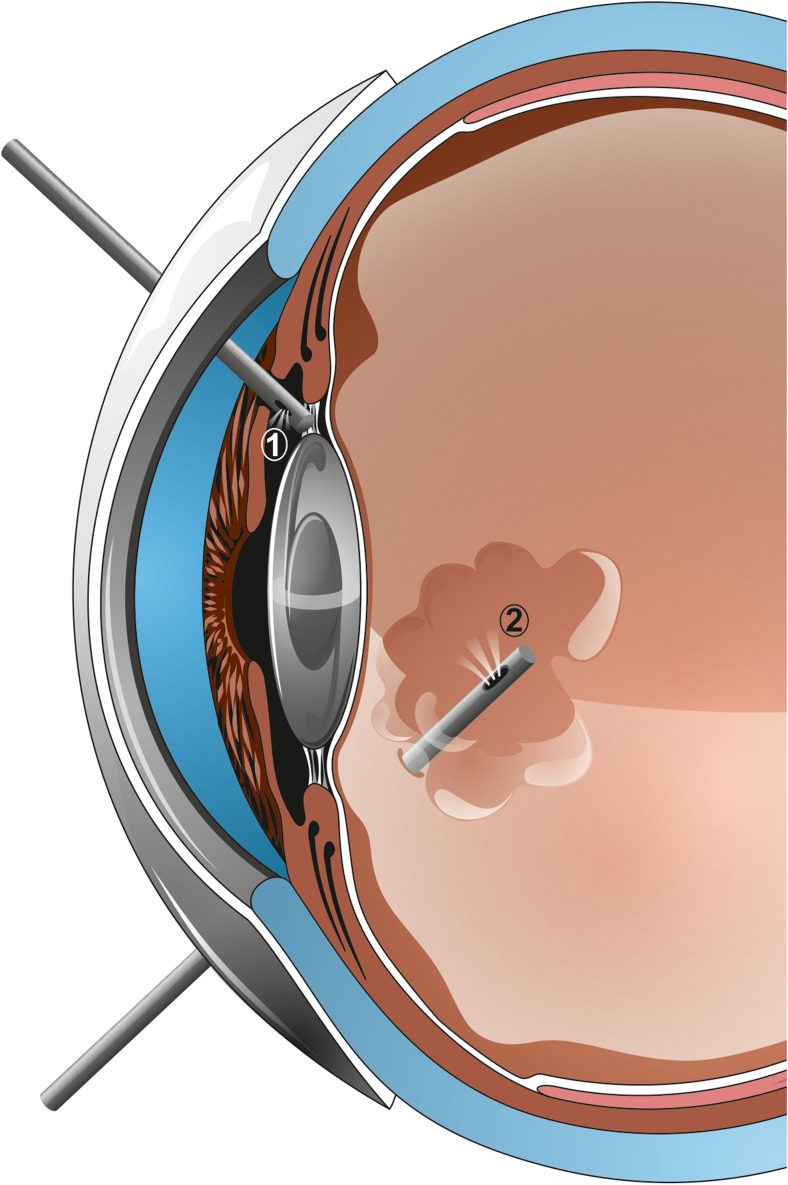
Recommended method of treatment. Vitreous decompression should be performed, preferably with hyaloido-capsulo-iridectomy. This treatment can be achieved with an anterior (*1*) or posterior (*2*) approach. In phakic eyes, concomitant cataract extraction would be desired

Thus, in several cases surgical treatment might be necessary. Certain authors stress that the greatest chance for permanent success is related to quick implementation of surgical treatment [51]. Furthermore, eyes with higher IOP and shorter axial length might be more likely to have a poor prognosis [52]. The fundamentals of the treatment are that evacuation of vitreous and aqueous humor from the vitreous cavity and establishment of communication with the anterior chamber helps to stop the vicious mechanism that eventually leads to increased IOP. Pars plana vitrectomy prevents aqueous accumulation inside the vitreous cavity, and it has been reported to be the treatment of choice for pseudophakic malignant glaucoma. Some authors suggest that a total vitrectomy, rather than partial removal of the anterior vitreous, is favorable [15]. However, this may not be enough to disrupt the cycle of malignant glaucoma, as it is postulated that all of the tissues (iris, lens capsule, zonule, and anterior vitreous) have to be removed to create a permanent passage between the anterior chamber and the vitreous cavity [10, 12, 20, 24]. (Fig. 2) Furthermore, Debrouwere et al. [24] emphasized that total vitrectomy was not effective in 66% of their patients unless an zonulectomy was added to the procedure. Part of the problem is that during conventional vitrectomy peripheral vitreous can hardly be completely removed, and that is why relapse rate may be very high [15]. Only total vitrectomy combined with zonulectomy, iridectomy, and capsulectomy has been described to be effective in 100% on larger groups of patients [12, 24]. In postoperative follow-up, it is important to maintain patency of newly created passages by using an Nd:YAG laser [12]. An alternative surgical treatment for pseudophakic malignant glaucoma suggested by anterior segment surgeons is zonulo-hyaloido-vitrectomy [14, 15, 17, 18]. In this procedure, the anterior vitrectomy is performed from the anterior chamber approach, through a tunnel within the iridectomy. It has been emphasized that this procedure is safer, as the iridectomy is done within visual sight, in contrast to a blind approach through the pars plana. The initial results of such a procedure are good, though recurrence might occur in up to 40% of cases. The reason is blockage of the channel by vitreous or fibrin [17]. In a recent Saudi Arabian study, the efficacy of vitrectomy combined with hyaloido-capsulo-iridectomy has been confirmed on a group of 69 eyes. A two-port pars plana vitrectomy with surgical microscope lighting can be as effective as a 3-port procedure [31]. Some authors underline that the removal of the anterior hyaloid face with capsulectomy is the key to resolving the symptoms of fluid misdirection syndrome, rather than debulking the vitreous [11, 31, 53]. Furthermore, small-gauge techniques might be as efficient as traditional 20-gauge vitrectomy [11].

Most of the cases described in literature do relate to pseudophakic eyes. If malignant glaucoma develops in a phakic eye most authors recommend concomitant vitrectomy and cataract extraction [10, 33]. Harbor et al. stress that lensectomy may be considered in eyes with substantial corneal edema or dense cataract, or when the anterior chamber does not deepen during vitrectomy [37]. Tsai et al. [33] reported better surgical outcome if an additional posterior capsulectomy was performed. Sharma et al. [10] suggest performing vitrectomy to reduce posterior pressure, followed by phacoemulsification and subsidiary vitrectomy followed by zonulo-hyaloidectomy. Chaundry et al. [54] claim that if one eye develops aqueous misdirection after surgery, prophylactic pars plana vitrectomy during cataract surgery of the contralateral eye may be beneficial.

## Conclusion

The *fluid misdirection syndrome* is a rare clinical condition characterized by an axially very shallow anterior chamber with the absence of suprachoroidal effusion or hemorrhage and no noticeable pathology of the iris–lens diaphragm. In all of the described cases, fluid becomes entrapped in PC. The positive pressure of the vitreous chamber does move the lens–iris diaphragm frontally, leading to angle closure and restricting spontaneous resolution. Hyperopia and lax zonular fibers/pseudoexfoliation can increase the risk for developing this syndrome. Furthermore, after an episode of malignant glaucoma in one eye, there is a high risk of a malignant course at the time of surgery or in the postoperative period [55]. This proves that all these disorders should be treated as one nosological syndrome.

We suggest the term *acute fluid misdirection syndrome* for the cascade of events during phacoemulsification surgery. The pathophysiology of *acute fluid misdirection syndrome* is based on inappropriate movement of balanced salt solution via the zonular fibers. This definitely better describes the nature of the syndrome rather than one of its signs. With that, understanding the pathophysiology will lead to unconditional and thought-out sorting out of the intervention.

We believe *chronic fluid misdirection syndrome* better describes the nature of *malignant glaucoma*. It is based on similar mechanisms of cilio-lenticular block of aqueous flow leading to the misdirection of aqueous posteriorly into or beside the vitreous gel, leading to the characteristic diffuse shallowing of the anterior chamber accompanied by a precipitous rise in intraocular pressure. It might seem unreasonable to consider *malignant glaucoma* a chronic process as, clinically, the marked increase of intraocular pressure occurs with some rapidity. However, it does occur gradually. The first symptom is often an improvement in near vision secondary to a myopic shift in refraction as the lens–iris diaphragm moves forward [16]. Furthermore, the inappropriate movement of aqueous and anterior chamber shallowing might be observed several days prior to intraocular pressure elevation. Therefore, malignant glaucoma can be difficult to detect early in its course before elevation in IOP occurs. The prognosis of this condition is good with currently available treatment modalities, and "malignant glaucoma" no longer deserves its historical name. It should be emphasized that using the current term leads to misunderstanding. It is necessary to explain to patients with malignant glaucoma that the term does not indicate a neoplastic process, and that glaucomatous damage to the optic disc is not always a consequence of the condition.

It is anticipated that using a clearly specified definition of *fluid misdirection syndrome* will enhance the validity of published data, assist in the identification of risk factors, and make it possible to establish an unified effective treatment for this dangerous condition.
